# Association between the Development of Hospice and Palliative Care and Government-Funded Research Priority: Taiwan-Based Example

**DOI:** 10.3390/healthcare10061125

**Published:** 2022-06-16

**Authors:** Ming-Chieh Cho, Po-Chin Yang, Yueh-Hsin Wang, Hsiao-Ting Chang, Ming-Hwai Lin

**Affiliations:** 1 Center for Geriatrics and Gerontology, Taipei Veterans General Hospital, Taipei 112, Taiwan; chuo60327@gmail.com (M.-C.C.); yhsin.wang@gmail.com (Y.-H.W.); 2 Department of Family Medicine, Taipei Veterans General Hospital, Taipei 112, Taiwan; michael00557@gmail.com (P.-C.Y.); htchang2@vghtpe.gov.tw (H.-T.C.); 3 School of Medicine, National Yang Ming Chiao Tung University, Taipei 112, Taiwan

**Keywords:** hospice and palliative care, field of investigation, government-funded research

## Abstract

In recent years, hospice and palliative care (HPC) has grown, developed, and changed in response to the humanistic and social needs for supporting those with incurable illnesses. As a relatively new discipline, research is needed in HPC, and the priority setting of research is essential to help direct finite resources to support research. To promote creative research in different fields including HPC, the Taiwan government subsidized institutions to conduct research. In this study, we obtained data from the Government Research Bulletin, an open-source online system containing complete information about government subsidized studies since 1993 to investigate the development of research priority in HPC in Taiwan. In total, 552 studies were recorded during 1993–2021, with a continued upward trend. An association was found between research priority and the promulgation of new HPC regulations. The type of diseases in research extended from cancer to all advanced chronic conditions. The increased diversity in out-of-hospital settings of palliative research was also observed. Numerous studies have focused on education, and the theme gradually shifted from “training and education for healthcare professionals” to “public education”. Here, the results may serve as a basis to understand the commonalities of research and enhance dialog in HPC research.

## 1. Introduction

Hospice and palliative care (HPC) has arisen due to the increased need for end-of-life care. As reported by the World Health Organization (WHO) in 2018, approximately 40 million people need HPC worldwide each year [1]. Studies have predicted that because of the change in population demographics, longer disease trajectories, and greater comorbidity, the demands and burden of palliative care are expected to escalate over the next decades [2].

HPC is a relatively new medical discipline. However, even though it has been advocated in global policy [3,4] and viewed as a basic human right [5], the investment in HPC research remains limited. When Cicely Saunders founded St Christopher’s Hospice in London in 1967, her vision was to build a hospice combining four important components, namely, (1) expert pain and symptom control, (2) compassionate care, (3) teaching, and (4) research [5]. Research is necessary for the development of medical disciplines including HPC. One of the most important reasons for research is to allow practitioners to deliver evidence-based treatment and care to improve outcomes of terminal illness [6]. Moreover, HPC research aims to present the core components of HPC, emphasizing its importance in policy and practice [7].

However, there are still some barriers for HPC research such as methodological, ethical and funding challenges. The heterogeneity of the HPC population represents challenges to research methodology, including study design, informed consent, and assessment. This is a vulnerable population in a situation complicated by physical decline and often debilitating symptoms, making HPC face moral and ethical challenges, especially on subjects who are seriously ill and/or dying [8,9]. Due to methodological and ethical challenges, identifying the priorities of HPC research can guide future research initiatives on specific topics [10]. There was a limited number of funding sources including government and private sources for HPC research. Previous studies have shown that the priority setting of research is an essential task to help direct finite resources to support research [11,12]. Identifying the priorities of HPC research helps researchers to know the current gaps of HPC research, and enable funders of research to target their funds to the priorities that matter [13].

Over the past decades, the world has witnessed Taiwan’s rapid development in HPC, and in 2015, the Economist Intelligence Unit ranked Taiwan sixth in the world and first in Asia with respect to HPC [14]. The development of HPC started in 1990 with the establishment of the first hospice ward in Mackay Memorial Hospital. In 1996, the Palliative Care Ward Governing Regulations and the Hospice Homecare Governing Regulations were announced, which allowed applications to the National Health Insurance Hospice Homecare Coverage Pilot Project [15]. In addition, 2000 was a milestone for HPC in Taiwan, as the Legislative Yuan passed the Hospice Palliative Care Act. It guaranteed the right to natural and dignified death for patients with terminal illness in Taiwan. The act allows physicians to discuss with the patients, who are in the terminal disease stage as diagnosed by two disease-specific specialists, their wishes regarding do-not-resuscitate decisions, hospice palliative care, and life-sustaining treatments (LST) [16]. Later, in 2006, hospice treatment was officially covered by National Health Insurance. In 2009, the National Health Insurance Administration announced hospice care for eight non-cancer diseases, which means that hospice care was no longer limited to cancer [17,18]. Afterward, the Patient Right to Autonomy Act was passed in 2016 and officially implemented in 2019; this was the first special law in Asia, signifying the milestone of patient autonomy and setting a precedent in Taiwan [19]. This act permits patients with the necessary decision-making capacity to make advance decisions about the complete or partial acceptance or refusal of LST and/or artificial nutrition and hydration under specific clinical conditions. Moreover, according to this law, when terminating, withdrawing, or withholding LST or artificial nutrition and hydration, the medical institution or physician should provide the patient with palliative care and other appropriate measures [20].

There were two systematic reviews about the research priority of palliative care published in 2014 and 2018, respectively. One of them gathered articles from 2005 to 2012, while another focused on studies published between 2008 and 2018 [11,21]. The results of these studies focused on the quantification of research. However, the change in trend of research priority areas over time was not discussed. To the best of our knowledge, there is an international paucity of research considering the priorities for HPC research, especially in Asia. Thus, this study aimed to investigate the development of research priority in HPC in Taiwan. Our findings may help understand the trend in research, identify gaps in the literature, and review the state of the science. Furthermore, our study may offer directions to researchers for further creative investigations.

## 2. Materials and Methods

### 2.1. Data Sources

The government of Taiwan subsidizes research projects that span one to three years. The amount of grant is given according to the number of years of study. The Government Research Bulletin (GRB) is an information system established by the government of Taiwan. GRB records include information about studies subsidized by the government since 1993. GRB contains six fields of investigation, including science, engineering, medicine, agriculture, humanities, and social studies. The number and percentage of studies in each field until 2021 were as follows: engineering, 865, 918, 518 (52%); medicine, 226, 928, 892 (14%); science, 220, 964, 723 (13%); social studies, 186, 326, 334 (11%); agriculture, 125, 685, 516 (8%); and humanities, 25, 585, 899 (2%). The implementation of GRB was supervised by the National Science Council, and it was built and maintained by the Ministry of Science and Technology. It contains the program number, titles of the studies in Chinese and English, year of subsidization, organizer, method, field of investigation, interval, subsidization price, principal investigator, keywords, and abstract. 

### 2.2. Study Design 

We extracted the total number of studies whose topics were about HPC from the GRB during the period from 1993 to 2021. A combination of HPC-related search terms was used, including “palliative”, “hospice”, “terminal care”, “end-of-life”, and “advanced decision”. The initial screening was performed by reviewing the title or abstract. We used the number of studies whose topics were about HPC to analyze the trend. We then compared these numbers to the total number of studies recorded from GRB over the same time. In addition, we used a basis point, representing one-one hundredth of one percent, to present the ratio. 

We classified HPC development in Taiwan into four periods by the promulgation of palliative-associated regulations or the change in National Health Insurance. Period 1 started in 1990 with the establishment of the first hospice ward and ended in 1999. Period 2 started in 2000 with the promulgation of the Hospice Palliative Care Act and ended in 2008. Period 3 started in 2009 with the announcement of hospice care for eight non-cancer diseases by the National Health Insurance Administration, and ended in 2015. Period 4 started in 2016 with the introduction of the Patient Right to Autonomy Act and extends to the present day. 

We extracted the numbers of studies (1) in various practice settings, (2) having specific types of diseases, (3) with different fields of investigation, and (4) whose research purpose was education, and then analyzed the trend of the total numbers and distribution in different categories.

- The annual mean of palliative-associated studies: refers to the mean of all newly opened studies in the same year.
- Practice settings: The “medical care unit” category included medical centers, metropolitan hospitals, local community hospitals, and any agency providing medical service. “Medical university” included universities having departments of medicine, nursing, pharmacy, physical therapy, occupational therapy, or public health, and “non-medical university” included other universities. “Other institutions” included all other institutions.
- Diseases: Diseases were categorized into “cancer”, “organ failure”, “dementia”, and “others”. Cancer included all kinds of malignancy regardless of its clinical stage. Organ failure included heart, respiratory, and renal failure. Patients who were using invasive ventilators and patients under hemodialysis or peritoneal dialysis were defined as having respiratory failure and renal failure, respectively.
- Field of investigation: Categories included “community”, “home care”, “long-term care unit”, “nursing home”, “emergency room”, and “intensive care unit”. We classified the studies by screening the title and abstract. If the above category was mentioned in the title or abstract, the study would be considered in that category.
- Education: Studies were included if their purpose was education and disciplines included “pedagogy” or “pedagogics of science”. Data regarding the discipline was obtained from the GRB.

### 2.3. Statistical Analysis

The data collection and analysis were performed with Microsoft Excel 2016 (Redmond, Washington, DC, USA) and presented in descriptive statistics. The categorical variables were presented in numbers and percentages.

## 3. Results

### 3.1. Changes in the Number and Ratio of Palliative-Associated Studies to Annual Total Studies from 1993 to 2021

**Figure 1:**
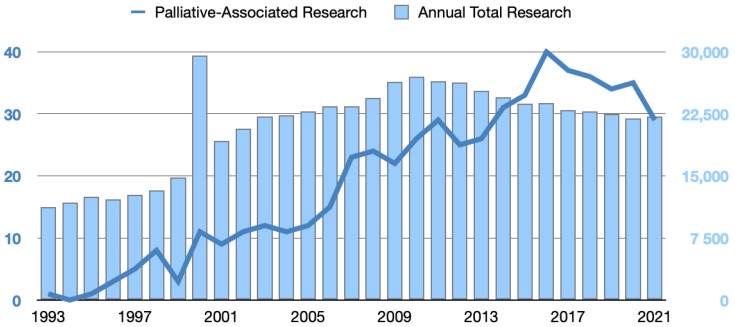
Changes in the number of palliative-associated studies to annual total studies from 1993 to 2021.

**Figure 2:**
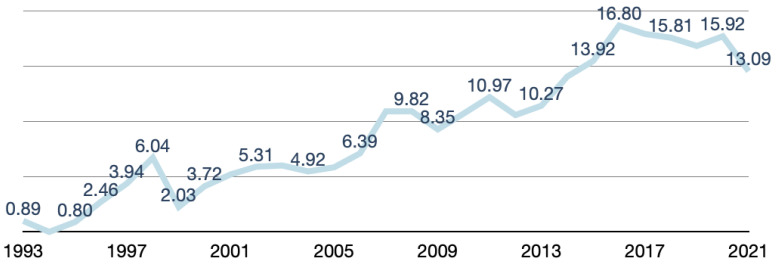
Changes in the ratio of palliative-associated research to annual total research in Taiwan from 1993 to 2021 (presented by basis point).

After screening, 552 studies were extracted from the GRB between 1993 and 2021. The number of palliative-associated studies increased steadily and reached its peak in 2016. Compared with one study conducted in 1993, 40 palliative-associated studies were conducted in 2016 (Figure 1). The ratio of palliative-associated studies to the annual total studies also increased yearly and reached its top in 2016. Compared with 0.89 basis points in 1993, the ratio of palliative-associated studies to the total number of studies was 16.80 basis points in 2016 (Figure 2). 

### 3.2. Changes in the Number of Palliative-Associated Studies and Annual Mean in Different Periods 

**Figure 3:**
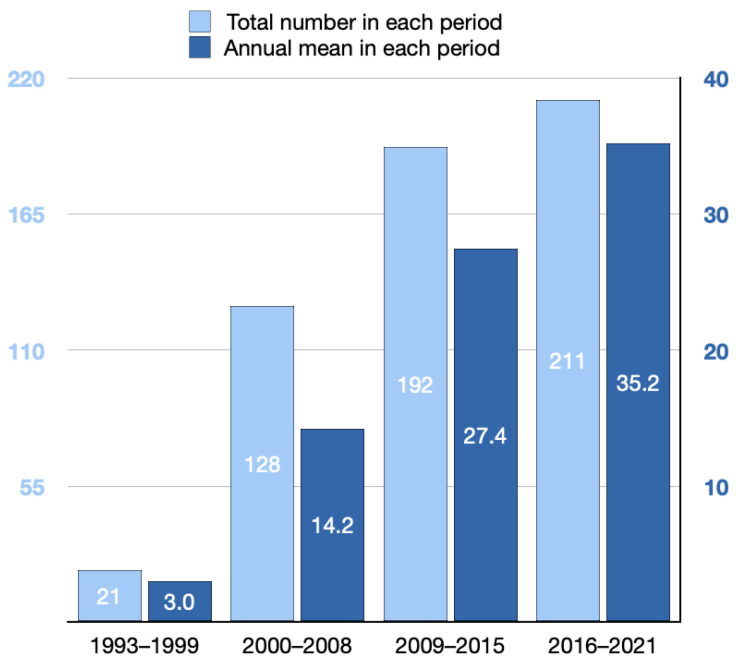
Changes in the number of palliative-associated research and annual mean in different periods.

The total number of palliative-associated studies increased in every period. The total number of studies in periods 1, 2, 3, and 4 were 21, 128, 192, and 211, respectively. The annual mean of palliative-associated studies increased every period, and the annual mean in periods 1, 2, 3, and 4 were 3, 14.2, 27.4, and 35.2, respectively (Figure 3).

### 3.3. Distribution of Studies in Various Practice Settings in Different Periods

The number of studies conducted in medical universities accounted for 67% of all studies in period 1, which gradually decreased to 52%, 56%, and 47% in periods 2, 3, and 4, respectively. 

As regards studies conducted in medical care units, 24% were conducted in period 1, which increased steadily to 28%, 28%, and 40% in periods 2, 3, and 4, respectively. 

**Figure 4:**
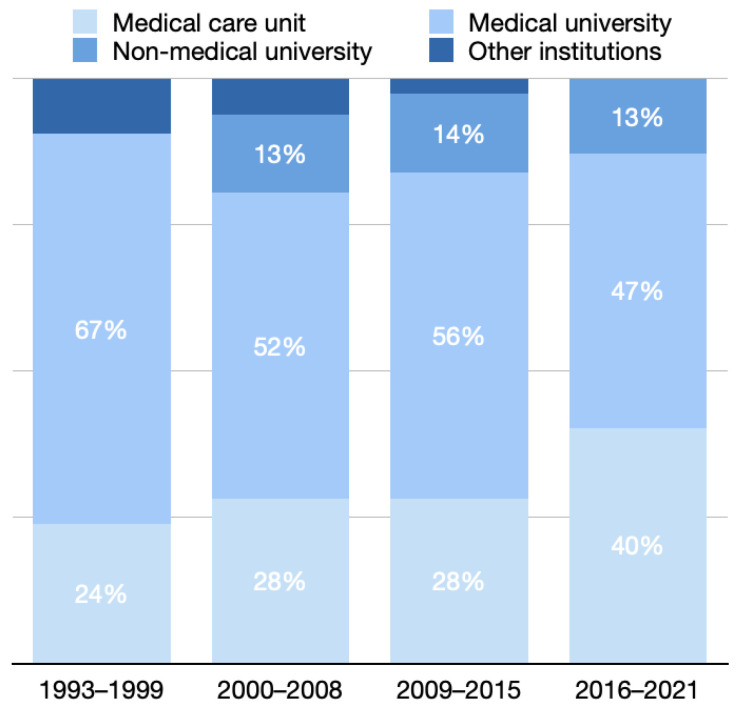
Distribution of studies in various practice settings in different periods.

No studies were conducted in non-medical universities in period 1, and in periods 2–4, the rate of studies conducted in non-medical universities varied between 13% and 14% (Figure 4). 

### 3.4. Distribution of Studies with Specific Types of Diseases in Different Periods

In the four periods, 8, 37, 52, and 61 studies targeted patients with specific diseases, and the annual mean values of each period were 1.1, 4.1, 7.7, and 10.2, respectively. 

Overall, cancer was the most common diagnosis of interest, followed by organ failure and dementia. In period 1, the type of disease in all studies was cancer (100%), which slightly decreased to 97% in period 2 and markedly decreased to 74% in period 3 and 46% in period 4. 

Among all studies, 13% and 11% of the studies focused on patients with dementia in periods 3 and 4, respectively. Moreover, 11% of the studies investigated patients with organ failure in period 3, which markedly increased to 36% in period 4.

**Figure 5:**
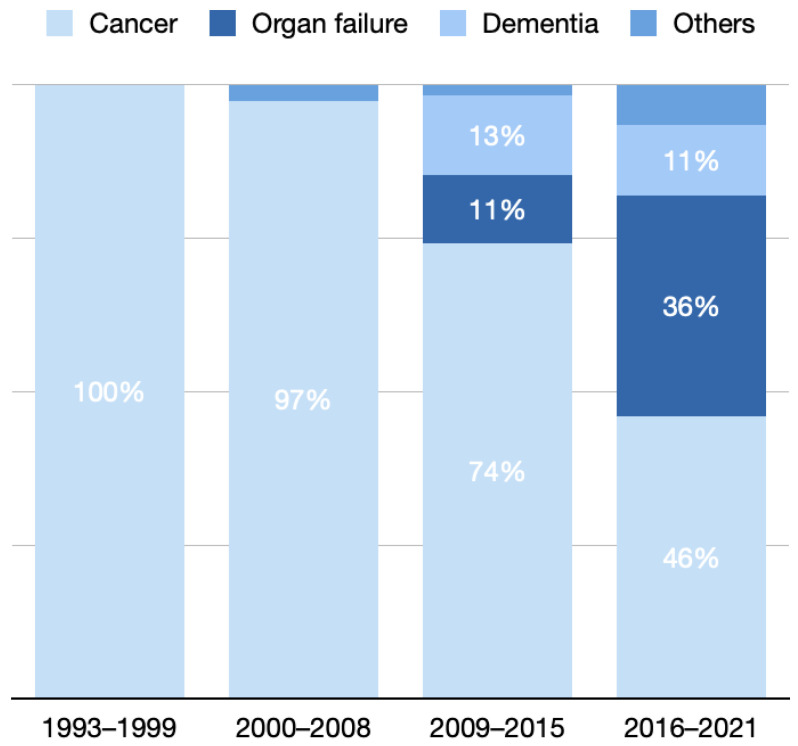
Distribution of studies with specific diseases of interest in different periods.

In period 2, 3% of the studies belonged to the “other diseases” category, including motor neuron disease. In period 3, 2% of the studies belonged to the “other diseases” category, including motor neuron disease and vegetative state. In period 4, 7% of the studies were in the “other diseases” category, including chronic obstructive pulmonary disease, sepsis, and vegetative state (Figure 5). 

### 3.5. Distribution of Fields of Investigation in Out-of-Hospital Settings in Different Periods 

In these four periods, 2, 16, 26, and 46 studies had out-of-hospital settings, and the annual mean values of each period were 0.3, 1.8, 3.7, and 7.7, respectively. In period 1, one study each took community and home as fields of investigation. Over time, the fields of investigation varied. 

In period 2, 38% of the studies with out-of-hospital settings took home care as a field of investigation; 31% took community; 13% took long-term care units and intensive care units; and 6% took nursing homes as a field of investigation. 

In period 3, 31% of the studies with out-of-hospital settings took long-term care unit as a field of investigation; 27% took nursing homes; 23% took the community; 15% took home care; and 4%, took intensive care units as a field of investigation. 

**Figure 6:**
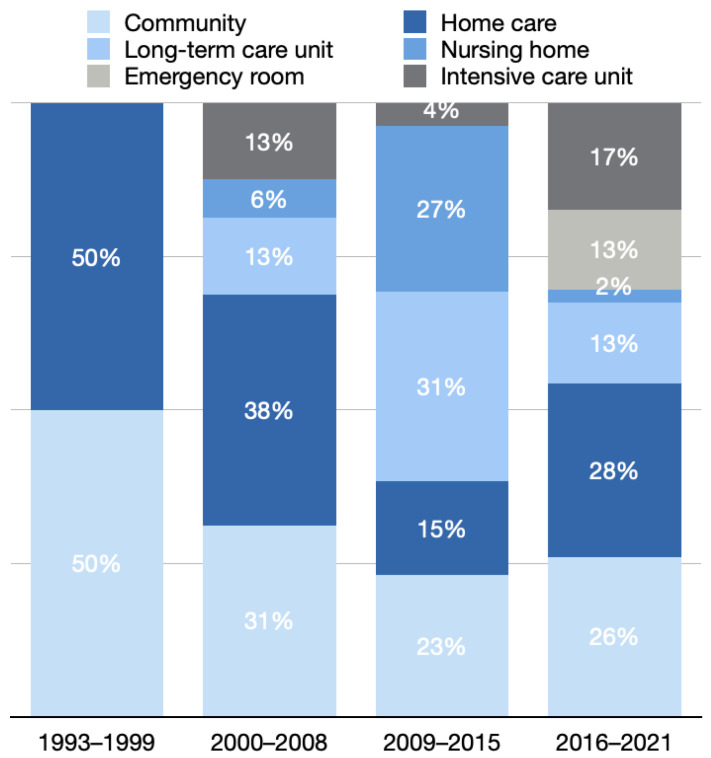
Distribution of fields of investigation in out-of-hospital settings in different periods.

In period 4, 28% of the studies with out-of-hospital settings took home care as a field of investigation; 26% took the community; 17% took intensive care units; 13% took long-term care and emergency rooms; and 2% took nursing homes as a field of investigation (Figure 6). 

### 3.6. Distribution of Receivers of Education in Different Programs

In the four periods, 3 (14.3%), 27 (21.1%), 53 (27.6%), and 48 (22.7%) studies focused on education, and the annual mean values of each period were 0.43, 3, 7.57, and eight, respectively. In period 1, all receivers of education were healthcare professionals. 

**Figure 7:**
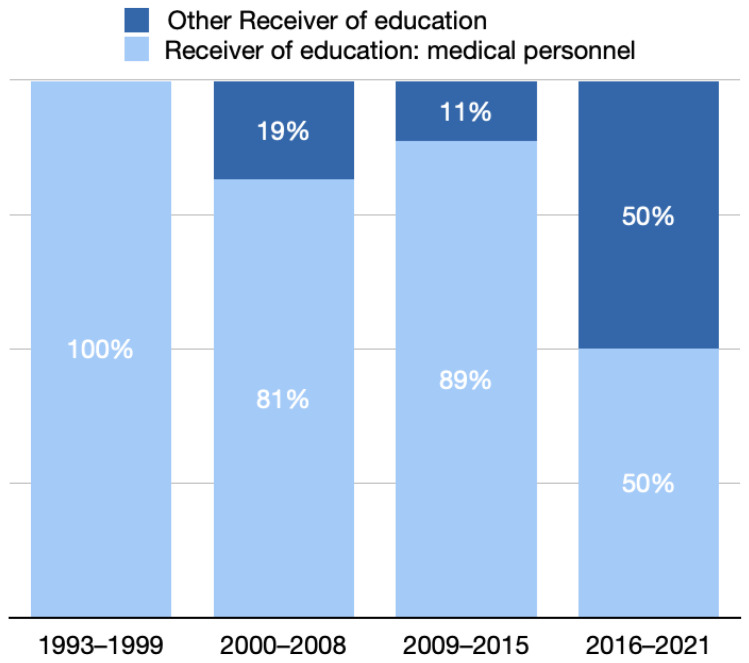
Distribution of receivers of education in different programs.

The number of studies whose receivers of education were healthcare professionals decreased to 81% and 89% in periods 2 and 3 and markedly decreased to 50% in period 4 (Figure 7). 

## 4. Discussion

### 4.1. Main Findings

The findings from this study provide an overview of Taiwan government-funded research in HPC in the past 29 years (1993–2021). The key findings revealed not only a continued upward trend in the research capacity in HPC in Taiwan but also the trends of research priority. More medical care units were investing in research. An association was found between “the surge of research in policy-related fields” and “the promulgation of new regulations”. As the concept of palliative care has changed, palliative-associated research priorities have also changed, not just focusing on people with cancer but also on people with all types of advanced chronic conditions. The increased diversity in out-of-hospital settings of palliative research was also observed. In addition to hospital wards, more studies were focusing on offering HPC in the community, home care, long-term care units, nursing homes, and emergency rooms and intensive care units. A trend was found, as more studies have focused on education, and the theme gradually shifted from “training and education for healthcare professionals” to “public education”. 

### 4.2. Types of Diseases of Research Shift from Cancer to Life-Limiting Illness 

In recent years, palliative medicine has grown, developed, and changed in response to the humanistic and social needs for supporting those with incurable illnesses and under conditions of suffering [22]. In the past, HPC was limited to those with terminal cancer [23].

At present, HPC has extended from its original focus on the care of patients with terminal cancer toward a wider perspective that includes the concept of “life-limiting” illness and early intervention, that is, to provide care to patients with all chronic diseases and conditions such as organ failure, dementia, motor neuron disease, and vegetative states. People with terminal cancer usually have a predictable prognosis and course of terminal decline. However, those with organ failure such as heart, lung, and renal failure may have a relative fluctuating decline and unpredictable prognosis, and they rarely have access to HPC [23]. This condition is also observed in individuals with frailty and dementia who need integrated clinical and social care. In 2002, the WHO provided the momentum to extend HPC in the illness trajectory [24]. In 2009, the National Health Insurance Administration of Taiwan announced hospice care for eight non-cancer diseases [17,18]. In a previous systematic review, a significant proportion of the research priority focused on patients with cancer, and it emphasized the need for palliative care research on patients with advanced cardiovascular, neurological, pulmonary, renal, and multiple comorbid diseases [11]. In our study, many studies have focused on patients with non-cancer illnesses. It not only identified the change of concept in HPC but also offered more evidence-based practice and knowledge. Because of the change in population demographics, longer disease trajectories, and greater comorbidity, the demands and burdens for HPC would escalate. More patients with different diseases under different conditions would need HPC. As a result, further research was required. 

### 4.3. Increased Diversity in the Field of Investigation 

HPC should be available not only in hospital wards but also in all settings. The concept of “HPC in all settings” extended its reach to people in hospitals, nursing homes, care homes, and most strategically, in the community. As out-hospital palliative service becomes more acceptable for patients and caregivers, numerous studies are focusing on out-of-hospital settings to provide better home-based or community-based HPC. One systematic review examined 10,235 publications and indicated the service model as one of the more popular research priority areas. The service model referred to care out-of-hospital settings and included the provision of out of hours care and home care services [12]. There is a need for better understanding and implementation of a model of care which identifies and delivers the palliative care needs in the community and other non-hospital settings. At the same time, besides home-based and community-based HPC, HPC became available in emergency rooms and intensive care units. 

Emergency providers frequently care for patients with a serious and life-limiting illness. Their providers play an integral role in initiating appropriate plans of care for patients in life-threatening conditions. The culture of emergency medicine to provide stabilization of acute medical emergencies is now shifting to a more patient-goal-centered culture [25]. There has been a huge movement to educate emergency physicians on end-of-life care and improve HPC in emergency medicine, and HPC starting in the emergency room can truly help patients and their families focus on their goal of care [26]. 

In the intensive care unit, patients with critical illness receive life-sustaining therapies to restore or maintain organ function. Recently, HPC in the intensive care unit is a widely discussed topic, and it is increasingly applied in clinics. Over the last two decades, intervention studies have explored how to better provide HPC together with critical care [27]. Therefore, further research is needed to better determine the institutional setting that can best provide HPC to patients and their families in both the emergency room and the intensive care unit. 

### 4.4. Increased Number of Studies Focusing on Education: From Healthcare Professionals to the Public

Previous systematic reviews identified that one of the palliative research priority areas was education and training. Health care provider education and training was recognized as a central component of delivering high-quality palliative care for non-palliative care specialists as the main group of HCPs, as well as non-hospital based providers [11,12].

Although HPC development has grown rapidly in Taiwan, people’s acceptance of the concept and practice of hospice care still needs improvement, not only for the general population but also for the service providers. Filial piety, as a core value in traditional Chinese culture, implied that children should take care of their aging parents for as long as possible until the end of their life. Moreover, it is unpropitious to talk about “death” in traditional Chinese culture. Death is an indispensable part of life, but the public’s awareness of death education is not sufficient. On the contrary, among physicians and nurses, there were gaps in understanding the concept of HPC, having the ability to communicate end-of-life issues, introducing HPC to patients and their families, confronting sociocultural issues, and being aware of the needs of HPC [28]. The development of HPC culture spurred the need for proper and formal training and public education. During the 1990s, education was extended in many countries, pre-graduate and postgraduate medical and nursing training programs were developed, training for other members of palliative teams was initiated, and the specialty of HPC was established [23]. Research evidence is crucial and essential to inform the content and implementation of training programs for healthcare professionals [29]. Furthermore, future research should identify the training needs of HPC to assess “the effect of training programs on provider practice and the outcomes” [30]. 

### 4.5. Limitations

This study has several limitations. First, the research source was limited to government-funded research from 1993 to 2021. We did not include studies funded by other resources or studies that were not subsidized. Second, we used the search words “hospice”, “palliative”, “end-of-life care”, “terminal care”, and “advanced decision”, which might not cover all HPC-related publications. Third, we counted only the number of studies but did not consider the study size, research method, or amount of subsidization. Finally, we did not completely read each study and therefore could not know the contents of these studies in order to conduct further analysis.

## 5. Conclusions

The government of Taiwan is dedicated to promoting the development of HPC by setting policies, pushing bills, and providing subsidies for research. These elements are mutually reinforcing. When subsidized by the government, these studies help inform the core components of HPC and help the government set policies and regulations. The findings of this study may serve as a basis to understand the commonalities of research and enhance the dialog in HPC research.

## Data Availability

Data are contained within the article.

## References

[1] World Health Organization (2018). Palliative Care. Fact Sheets.

[2] Sleeman K.E., de Brito M., Etkind S., Nkhoma K., Guo P., Higginson I.J., Gomes B., Harding R. (2019). The escalating global burden of serious health-related suffering: Projections to 2060 by world regions, age groups, and health conditions. Lancet Glob. Health.

[3] Powell R.A., Mwangi-Powell F.N., Radbruch L., Yamey G., Krakauer E.L., Spence D., Ali Z., Baxter S., De Lima L., Xhixha A. (2015). Putting palliative care on the global health agenda. Lancet Oncol..

[4] Harding R., Higginson I.J. (2014). Inclusion of end-of-life care in the global health agenda. Lancet Glob. Health.

[5] Mathew A., Cowley S., Bliss J., Thistlewood G. (2003). The development of palliative care in national government policy in England, 1986–2000. Palliat. Med..

[6] Preston N.J., Payne S.A., Todd C. (2009). Conducting research in palliative care patients: Burden or an opportunity?. Int. J. Palliat. Nurs..

[7] Curie M. From Research to Policy and Practice: Marie Curie Annual Research Impact Report, 2014/2015. https://www.mariecurie.org.uk/globalassets/media/documents/research/publications/research-impact-report-2014-15.pdf.

[8] Dean R.A., McClement S.E. (2002). Palliative care research: Methodological and ethical challenges. Int. J. Palliat. Nurs..

[9] Abernethy A.P., Capell W.H., Aziz N.M., Ritchie C., Prince-Paul M., Bennett R.E., Kutner J.S. (2014). Ethical Conduct of Palliative Care Research: Enhancing Communication Between Investigators and Institutional Review Boards. J. Pain Symptom Manag..

[10] Higginson I. (2016). Research challenges in palliative and end of life care. BMJ Support. Palliat. Care.

[11] Riffin C., Pillemer K., Chen E.K., Warmington M., Adelman R.D., Reid M.C. (2015). Identifying Key Priorities for Future Palliative Care Research Using an Innovative Analytic Approach. Am. J. Public Health.

[12] Knaul F.M., Farmer P.E., Krakauer E.L., De Lima L., Bhadelia A., Jiang Kwete X., Arreola-Ornelas H., Gómez-Dantés O., Rodriguez N.M., Alleyne G.A.O. (2018). Alleviating the access abyss in palliative care and pain relief-an imperative of universal health coverage: The Lancet Commission report. Lancet.

[13] Best S., Tate T., Noble B., Eley J., Black J., Stockton M., Cheesley A., Berry L., Loftus R., Dechamps A. (2014). The palliative and end of life care priority setting partnership (PEOLCPSP): Determining evidence uncertainties from the perspective of the end user of research. BMJ Support. Palliat. Care.

[14] The Economist Intelligence Unit Limited (2015). The 2015 Quality of Death Index Country Profiles.

[15] Cho C.Y. (2018). From cure to care: The development of hospice care in Taiwan. Hosp. Palliat. Med. Int. J..

[16] Hospice Palliative Care Act. https://law.moj.gov.tw/ENG/LawClass/LawAll.aspx?pcode=L0020066.

[17] Chen R.C. (2016). A personal journey in Taiwan’s hospice palliative care movement. BAOJ Palliat. Med..

[18] Ming-Hui L., Tzeng-Ji C. (2009). Current Developments in Hospice Treatment in Taiwan.

[19] Patient Right to Autonomy Act. https://law.moj.gov.tw/ENG/LawClass/LawAll.aspx?pcode=L0020189.

[20] Chen R.C. (2017). Hospice treatment philosophy, history, working models, current status and future prospects. Lien Found. Spirit. Care Curric..

[21] Hasson F., Nicholson E., Muldrew D., Bamidele O., Payne S., McIlfatrick S. (2020). International palliative care research priorities: A systematic review. BMC Palliat. Care.

[22] Pastrana T., Junger S., Ostgathe C., Elsner F., Radbruch L. (2008). A matter of definition—Key elements identified in a discourse analysis of definitions of palliative care. Palliat. Med..

[23] WHPCA (2017). Building Integrated Palliative Care Programs and Services.

[24] Moroni M., Bolognesi D., Muciarelli P.A., Abernethy A.P., Biasco G. (2011). Investment of palliative medicine in bridging the gap with academia: A call to action. Eur. J. Cancer.

[25] Chan G., Bryant E.N., Lamba S., Weissman D.E., Quest T.E., Knox T.H. (2011). Clinical Practice Guidelines: A Technical Assistance Resource from the IPAL-EM Project [Internet].

[26] Mierendorf S.M., Gidvani V. (2014). Palliative care in the emergency department. Perm. J..

[27] Rebecca A., Curtis J.R., Judith E. (2014). The changing role of palliative care in the ICU. Crit. Care Med..

[28] Tay J., Compton S., Phua G., Zhuang Q., Neo S., Lee G., Wijaya L., Chiam M., Woong N., Krishna L. (2021). Perceptions of healthcare professionals toward palliative care in internal medicine wards: A cross-sectional survey. BMC Palliat. Care.

[29] Canadian Cancer Research Alliance (2017). Pan-Canadian Framework for Palliative and End-of-Life Care Research.

[30] Pillemer K., Chen E.K., Riffin C., Prigerson H., Reid M.C. (2015). Practice-based research priorities for palliative care: Results From a research-to-practice consensus workshop. Am. J. Public Health.

